# Assessment of Malaria Microscopy Competency at Primary Health Institutions in the Chongqing Municipality

**DOI:** 10.3389/fmed.2021.602442

**Published:** 2021-03-15

**Authors:** Luo Fei, Zhou Shuang, Yuan Yi, Li Shan-Shan, Tan Yan, Xu Jing-Ru, Zhou Yang

**Affiliations:** Chongqing Center for Disease Control and Prevention, Chongqing, China

**Keywords:** malaria, microscopy, competency assessment of malaria microscopy, plasmodium species identification, Chongqing municipality

## Abstract

**Background:** In April 2019, Chongqing passed the national malaria elimination assessment. However, around 30 imported malaria cases are still being reported every year, and *Anopheles sinensis* was widely distributed in Chongqing, meaning the risk of malaria resurgence still exists. Early diagnosis and treatment for malaria cases are effective measures to prevent malaria resurgence. The primary health institutions are the first station where potential malaria cases may seek treatment. The competency with which primary health institutions diagnose malaria will directly affect the timeliness of malaria diagnosis. Nowadays, most primary health institutions in Chongqing use microscopy to confirm malaria cases. This study assessed the microscopy competence of primary health institutions, studied and analyzed the results, and provided a scientific basis for malaria prevention and control after malaria elimination in Chongqing.

**Methods:** According to the stratified sampling principle, four plasmodium microscopy technicians (156 in total) were selected from each of the 39 districts/counties of Chongqing to test the plasmodium microscopy competence. Descriptive statistical analysis, correlation analysis, spatial self-correlation analysis, and ROC curve analysis were carried out on the test results.

**Result:** The average of the technicians' test scores was 4.33 ± 0.47 (min: 3, mid: 4.5, max: 5); The spatial clustering of the scores was significant (MoranI = 0.338, *Z* = 3.618, *P* < 0.01). The test scores were positively correlated with the “level of work institutions” (*R* = 0.21, *P* < 0.01) but were negatively correlated with “age” (*R* = −0.31, *P* < 0.01). The highest Sensitivity of the technicians' microscopy was in qualitative diagnosis (98.92%, CI: 98.00–99.69%). The Delong's test showed that the diagnostic efficiency of the technicians' microscopy to *P. falciparum* was the best (*P* < 0.01), however to *P. ovale* was the worst (*P* < 0.01).

**Conclusion:** The microscopy technicians in primary health institutions in Chongqing have good microscopy competency in qualitative diagnosis, but there were deficiencies in the identification of plasmodium species. Township level health institutions in Central China have weaker microscopy than those in other areas.

## Background

Malaria is one of the most serious parasitic diseases. According to WHO, there were 2.28 million malaria cases and 405,000 deaths worldwide in 2018, and 93% of the cases were in Africa (1). In 2019, a total of 2,674 malaria cases were reported in China, including 19 deaths. The median interval from onset to diagnosis was 2 days (2).

Chongqing has launched the Malaria Elimination Plan in 2010 which includes implementation of comprehensive malaria control and prevention activities (3). After 10 years of implementation, Chongqing passed the national malaria elimination assessment in April 2019. In the last decade, Chongqing has reported about 30 imported cases annually and 125 imported cases from Africa, accounting for 84.46%. After achieving the goal of malaria elimination, the next objective for Chonqing is to prevent malaria resurgence caused by imported cases and the occurrence of severe death cases. In order to achieve this, it is necessary to make sure that no malaria case be misreported in Chonqing. There are about 30 imported malaria cases still reported every year in Chonqing, and Anopheles sinensis strain is widely distributed in Chongqing, meaning the risk of malaria resurgence still exists (4, 5). Early diagnosis and treatment for malaria cases are effective measures to prevent malaria resurgence and the occurrence of severe cases and deaths (6–9). The primary health institutions are the first station where potential malaria cases may seek treatment. The malaria diagnosis competency of the primary health institutions will directly affect the timeliness of malaria diagnosis (10, 11). In 2019, 38 malaria cases and two deaths were reported. The median interval from onset to diagnosis was 2 days; only 39.19% of the cases were within 24 h (12). Microscopy of plasmodium is an important approach to confirm patients and is also the gold standard for the diagnosis of malaria (13). A well-trained technician may realize high sensitivity and specificity when using microscopy to detect Plasmodium from blood slides whose parasitemia threshold could be 50 count/UL (13, 14). However, the efficiency of malaria microscopy is affected by the quality of the microscope and the competency of the technician, both of which have a great influence on the timely and accurate diagnosis of malaria cases (15–17). Nowadays, most primary health institutions in Chongqing use microscopy to confirm malaria cases. Therefore, it is important to know the current status of malaria microscopy in the primary health institutions. This study carried out the malaria microscopy competency test on technicians in primary health institutions in Chongqing to understand the current status of their competency with malaria microscopy in the qualitative analysis of plasmodium blood slides and plasmodium species identification. The test also analyzed related factors affecting their performance, so as to accurately improve the malaria diagnosis competency of primary health institutions, provide a scientific basis for the improvement measures of malaria early diagnosis, and reduce the occurrence of severe and deadly malaria cases.

## Methods

### Study Area and Objects

Chongqing is located in southwest China in the upper reaches of the Yangtze River. It covers an area of 82,400 km^2^. There are 38 districts/counties and one economic development zone in Chonqing. Its permanent population is 31.24 million, and the urbanization rate is 66.8%. The landform is mainly composed of hills and mountains, of which 76% are mountains. It has a subtropical monsoon humid climate. The Yangtze River runs through the whole territory, with a flow path of 691 km, and joins the Jialing River and Wujiang River (18).

The first step in the national “*Malaria Elimination Assessment Program (2014 Edition)”* and the principle of stratified sampling means that all 39 district/county level CDCs have participated in the study. Two to three clinical medical institutions were randomly selected according to the actual situation of each district and county. The second step involved one microscopic technician being randomly selected from 39 CDCs, and three microscopic technicians being randomly selected from the selected health institutions, making up four people from each district/county to participate in the malaria microscopy competence test.

### Design of Microscopy Competence Test

The malaria microscopy competency single blind tests were conducted separately in each district/county from 2014 to 2017 with one bind. There were four sets of standard blood slides for tests that were provided by the Malaria Diagnostic Reference Laboratory of Chongqing CDC. The blood slides were made in line with the requirements of the national “*Malaria Elimination Assessment Program (2014 Edition)”* and the WHO “Operating Manual for Microscopy and Evaluation of Plasmodium” (15). Each set of standard blood slides was composed of three *P. falciparum* slides classified by the density of plasmodium with three levels that were high (≥84,000 counts/ul), medium (≥2,800 counts/ul), and low (≥500 counts/ul), one *P*. vivax slide, one *P. ovale* slide, and one negative slide. The parasitemia of four *P. vivax* slides and four *P. ovale* slides were 10,072–13,280 counts/ul and 2,293–3,072 counts/ul. The positive slides were made by the malaria patients' blood which was stored in the sample bank of Chongqing CDC. The negative slides were made using a healthy person's blood. All of the blood samples were confirmed by NEST-PCR and the blood slides were double checked by technicians who had acquired a WHO malaria microscopy competency level one certification to guarantee correct diagnosis. Only the blood slides with the same diagnosis of NEST-PCR and microscopy were used in this research. When the microscopy competency test was conducted, each technician would randomly select one set, and took five blood films from the set. The microscopy of plasmodium must be completed within 1.5 h. Five points were awarded if all were correct. One point was deducted for one qualitative error and 0.5 point was deducted for one species error.

### Statistical Analysis

The test results and patient information were collected and sorted, and a database in the Excel 2016. R.4.02.application was used for statistical analysis. The districts and counties of Chongqing were divided into five geographical “urban areas”: “Western Chongqing,” “Central Chongqing,” “Southern Chongqing,” “Northeast Chongqing,” and “Southeast Chongqing.” One factor analysis of variance was conducted for the scores. The judgment results of each blood film were recorded and scored according to the design principles. The “Kendall” correlation analysis was carried out on the variables of “score,” “gender,” “age,” “working unit level,” and “professional qualification grade” of the technicians. The average scores of 39 districts/counties were calculated, and the “overall self-correlation” analysis was done with ArcMpa16.0, and the “Clustering Distribution Map” was plotted.

The qualitative diagnosis of microscopy and sensitivity, specificity, positive predictive value, negative predictive value, and Youden index in different plasmodium determinations were calculated and the ROC curve was plotted, which was to be paired and compared using “Delong's test.” When calculating the diagnostic indexes of different species, the positive determination of the blood film of the species was considered valid, while the negative or other species determination was considered invalid.

## Result

### General

A total of 156 microscopic technicians from 39 districts/counties participated in the test, including 56 males (35.90%) and 100 females (64.10%), with a male to female ratio of 0.56; the age of the technicians was between 19 and 64 years and the average age was 34.89 ± 8.91, of which 56 (35.90%) were under 30 years old. 85 (54.50%) were at County-level medical institutions; 78 (50%) had junior technical qualifications. In the assessment results, there were 13 (8.30%) technicians with one qualitative error, 75 (48.10%) with one species identification error, 43 (27.60%) with two species identification errors, and eight (5.10%) with three species identification errors. The average score was 4.33 ± 0.47; the lowest was 3, the highest was 5, and the median was 4.5 (Table 1).

**Table 1:** Summary of the subjects.

**Items**		**Count**	**Proportion**
Gender	Male	56	35.90%
	Female	100	64.10%
Age	34.89 ± 8.91 (Range: 19–64, Mid: 33)		
Age group	<30	56	35.90%
	≥30	54	34.62%
	≥40	46	29.49%
Work units	County CDC	40	25.64%
	County hospitals	85	54.49%
	Town hospitals	31	19.87%
Technical qualifications	Junior	78	50.00%
	Senior	11	7.05%
	Intermediate	67	42.95%
Total of Qualitative errors slides	0	143	91.67%
	1	13	8.33%
Total of species errors slides	0	30	19.23%
	1	75	48.08%
	2	43	27.56%
	3	8	5.13%
Scores	4.33 ± 0.47 (Range: 4.33–5, Mid: 4.5)		
	3.0	3	1.92%
	3.5	15	9.62%
	4.0	43	27.56%
	4.5	67	42.95%
	5.0	28	17.95%

### Regional Distribution

**Figure 1:**
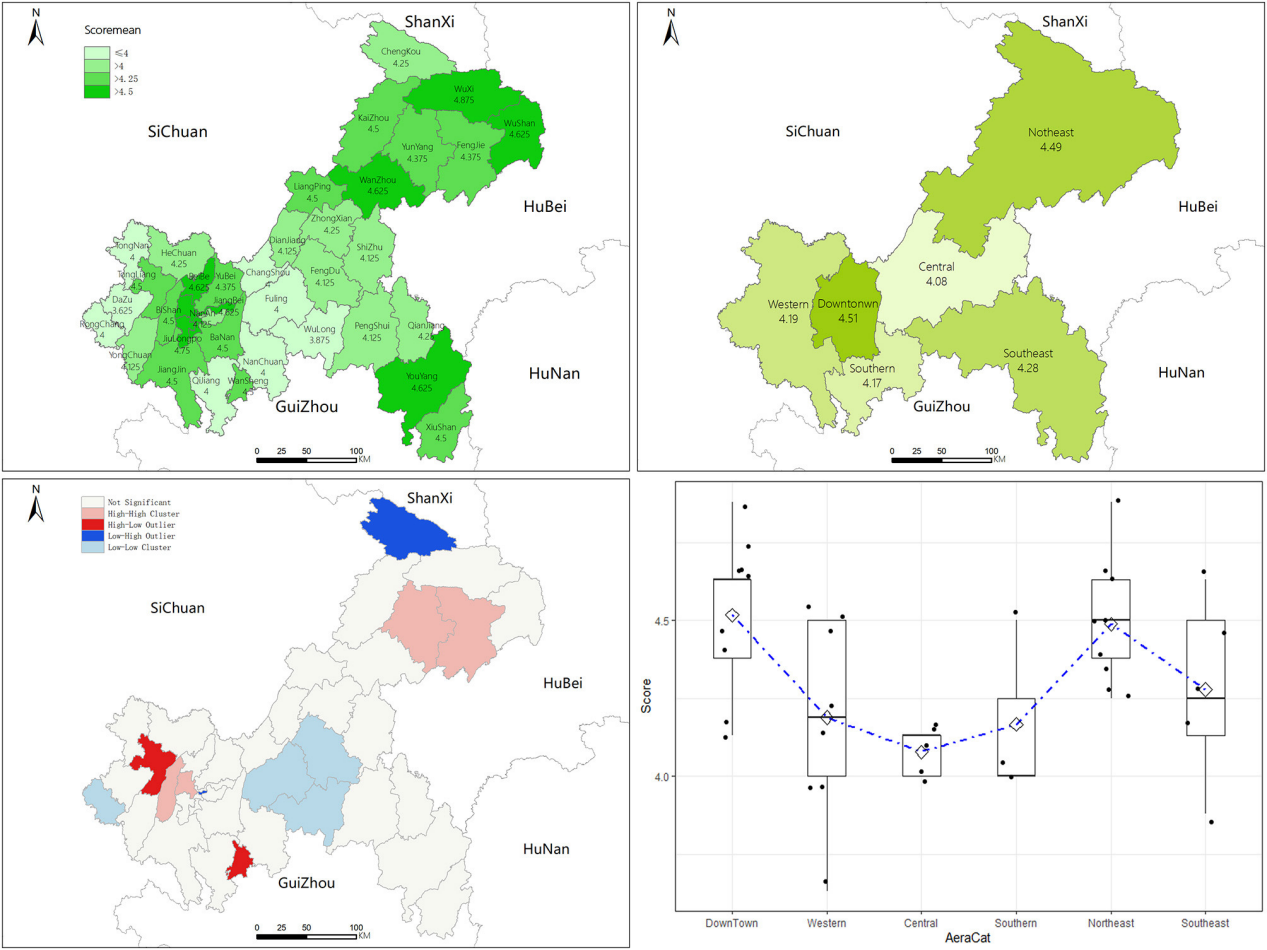
Geographic information analysis map and statistical analysis chart of the test scores.

Among the 38 districts/counties and one economic development zone, Shapingba District and Wuxi County got the highest average score of 4.875, followed by Jiulongpo District with 4.75. Dazu District got the lowest score of 3.625. The results of global spatial self-correlation analysis showed that the scores had significant spatial clustering (MoranI = 0.338, *z* = 3.618, *P* < 0.01). From the clustering distribution map based on the average scores of all districts/counties, we can see that the highest scores occurred in Western Chongqing, the urban area, and Northeast Chongqing, and the low scores were concentrated in the central part of Chongqing. The results showed that there were significant differences in scores among the geographical distribution regions (*F* = 3.475, *P* = 0.012). There was no significant difference between the “Urban area” (4.51) and “Northeast Chongqing” (4.49) (*F* = 0.728, *P* = 0.41), and the lowest score was *4.08* in Central Chongqing, which was much lower than that in other regions (*P* < 0.05), see Figure 1.

### Diagnostic Competence

The sensitivity of microscopy was the highest in qualitative diagnosis (98.92%, CI: 98.00–99.69%) and the lowest in *P. ovale* diagnosis (15.94%, CI: 8.70–24.64). The highest specificity of diagnosis occurs in *P. ovale*, 96.20% (96.20%, CI: 94.66–97.61%) and the lowest in *P. vivax* (77.22%, CI: 73.87–80.74%). The negative predictive value and positive predictive value of qualitative diagnosis were the highest, respectively, 94.70 and 99.07%. The lowest negative predictive value was 78.54% for *P. falciparum* and 28.95% for *P. ovale*. The highest Youden Index was 0.9434 for qualitative diagnosis and 0.1214 for *P. ovale*, see Table 2.

**Table 2:** Indexes of the technicians' microscopy.

**Items**	**Sensitivity (%)**	**95% CI of sensitivity (%)**	**Specificity (%)**	**95% CI of specificity**	**NPV (%)**	**PPV (%)**	**Youden index**
Qualitative	98.92	(98.00–99.69)	95.42	(91.60–95.42)	94.70	99.07	0.9434
*P. f*	75.57	(71.53–79.35)	92.69	(90.08–95.04)	78.54	91.46	0.6826
*P. v*	79.23	(72.68–84.70)	77.22	(73.87–80.74)	92.38	51.60	0.5645
*P. o*	15.94	(8.70–24.64)	96.20	(94.66–97.61)	92.18	28.95	0.1214

**Figure 2:**
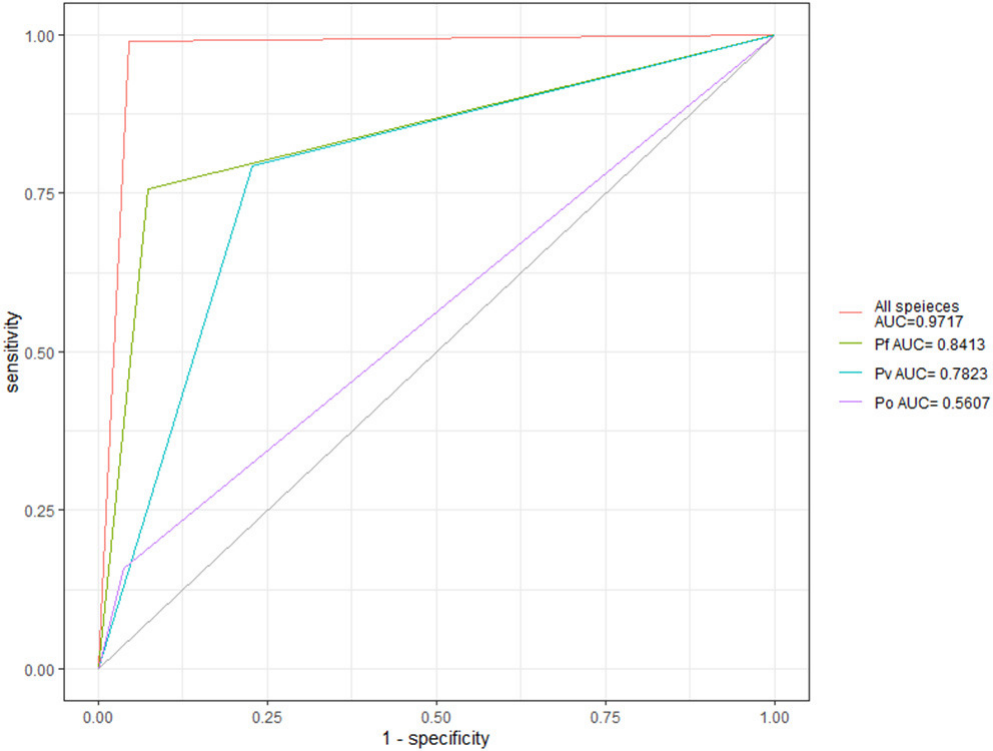
The technicians' microscopy ROC curve for different plasmodium species.

The area under ROC curve for qualitative diagnosis, diagnosis of *P. falciparum, P. vivax*, and *P. ovale* were, respectively, 0.9717, 0.8413, 0.7823, and 0.5607 for technician microscopy. See Figure 2.

Delong's test was used to compare the ROC curves microscopy for diagnosis of different plasmodium species. The results showed that the diagnostic efficiency of *P. falciparum* was significantly higher than that of other species (*P* < 0.01), see Table 3.

**Table 3:** DeLong's test on the technicians' microscopy ROC.

**DeLong' test**	**Area under curve (AUC)**	***Z-*value**	***P***
*P. f* vs. *P. v*	0.8412 vs. 0.7823	2.75	<0.01
*P. f* vs. *P. o*	0.8412 vs. 0.5607	10.87	<0.01
*P. v* vs. *P. o*	0.7823 vs. 0.5607	7.81	<0.01

### Correlation Analysis

**Figure 3:**
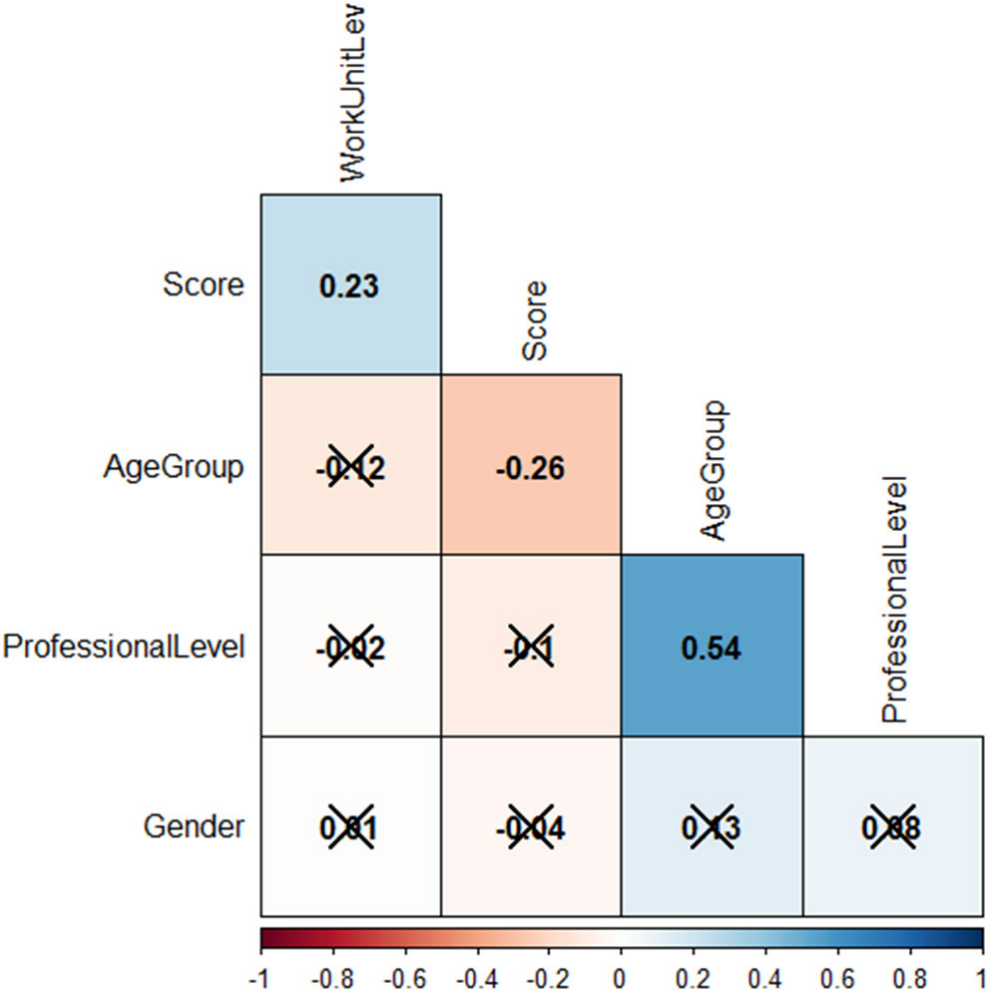
Correlation analysis matrix to the technicians. × indicates *P* > 0.05.

The variables were encoded: Gender: 0 = female, 1 = male; units: 1 = township medical institutions, 2 = county-level medical institutions, 3 = county-level CDC; qualification: 1 = junior, 2 = intermediate, 3 = senior; age group: 1 = under 30, 2 = 30–40, 3 = above 40. “Kendall” correlation analysis showed that the score of technicians was positively correlated with unit level (*r* = 0.21, *P* < 0.01) and negatively correlated with age (*r* = −*0.31, P* < 0.01). There was a positive correlation between technical qualification and age (*r* = 0.5, *P* < 0.01), see Figure 3.

## Discussion

The results of this study showed that the overall microscopy competency was the best in qualitative diagnosis, and the sensitivity and specificity microscopy were at a high level. There were differences in scores amongst different regions. The scores in the urban area and Northeast Chongqing were significantly higher than those in other regions, and the lowest score was in Central Chongqing. The reason could be that the number of malaria cases reported in the urban area of Chongqing accounts for about 60% of the total cases in Chongqing (12, 19, 20), and the economic, social, and health development indexes in the urban area are also higher than those in other areas (21–23). Although the quality of malaria microscopy is more affected by the competency of the technician (14, 24, 25), the local economic and social development and the number of imported malaria cases may also be factors affecting the microscopy competency of the primary health institutions.

The results showed that the qualitative diagnostic efficiency of plasmodium microscopy was the highest, followed by *P. falciparum*. The scores of the identification of *P. vivax* and *P. ovale* were quite low; the positive predictive value was only 51.60 and 28.95%. Morphological identification of P. vivax and *P. ovale* by microscopy has always been difficult, and requires rich experience and technical training from the technician (26, 27). That's why, in this study, there were many misjudgments in the identification between the two. The treatments for *P. vivax* and *P. Ovale* are basically identical (6, 28); although the confusion between the two species will not have a significant impact on the treatment of patients, the earlier detection, earlier diagnosis, and earlier treatment of imported *P. vivax* malaria are of great significance to prevent the malaria resurgence of imported cases after the elimination of malaria in China (2, 29). At present, there is little research on the transmission competency of *A. sinensis* to *P. ovale*, and there is no clear evidence that imported *P. ovale* can exist in local *Anopheles sinensis* and infect humans. The primary doctors may neglect management of imported *P. ovale*; if *P. vivax* were misdiagnosed as *P. ovale*, the risk of malaria resurgence will increase. In recent years, the number of imported cases of *P. ovale* malaria cases has increased year on year (20, 29, 30), which is related to the establishment of malaria reference laboratories nationwide to carry out PCR reviews for each reported case since the implementation of the malaria elimination action plan. Compared with other detection methods, PCR detection can more accurately distinguish various types of plasmodium species (25, 27, 31–33). In 2012, the malaria reference laboratory was established in Chongqing CDC. All malaria cases reported by health institutions in Chongqing need to collect blood samples and send them to the reference laboratory for PCR review (3, 34). Therefore, a malaria reference laboratory is an important technical support for accurate diagnosis of malaria cases and an important means to prevent the transmission of imported malaria.

In the correlation analysis of technician's personal characteristics, it was found that the higher the level of work unit, the higher the score. Technicians from the township level health institutions had lower scores, and their microscopy competencies were relatively lower. The study also found that older technicians scored lower. Under normal circumstances, the technician's competency of plasmodium microscopy will increase with age; rich microscopy experience leads to better competence (13, 26, 35). However, there were no local malaria cases reported in Chongqing from 2011, and there were only about 30 imported cases each year in last decade. Seventy percentage of the cases were reported by provincial, municipal, and county-level health institutions, and only about 8% of the cases were reported in the primary township level health Institutions (12). This situation makes it difficult for doctors and laboratory staff of township health institutions to receive malaria patients or see positive blood film in field work. As the age of the technician increases, their experience will not increase due to a lack of practice, on the contrary, it will be forgotten. In recent years, based on the principle of training young malaria prevention and control personnel, Chongqing has held a parasite microscopy competition every year, requiring all participants dispatched be under 35 years (36, 37), which indirectly leads to the training of primary malaria microscopy technicians focusing on young people. The likelihood of receiving malaria patients at the township level is low, and the microscopy technician may easily quit (22, 38). These factors will lead to the decline of the microscopy competence of primary microscopy technicians. At the same time, with the increase of age, the diagnostic competency of microscopy technicians will decrease. In contrast, Rapid Diagnostic Test (RDT) is more convenient and faster than plasmodium microscopy in the areas lacking equipment, technicians, and technologies, and has lower requirements on the technicians' competence (33, 39), so is more suitable to be widely used in primary health institutions in Chongqing.

### Study Limitation

Due to limitations imposed by funds and manpower, only four microscopy technicians from three kinds of health institutions from each district/county were selected to participate in the test, and the sampling volume was small. Due to the lack of good blood samples *P. malariae* was not included in the test. The competency tested was limited to the microscopy technician's competence of the blood film reading, not the competence of film preparation. In this study, we failed to collect complete data for utilizing social, economic, and health resources, and did not conduct further study on the influence of these factors on the competency of microscopy in various regions.

## Conclusions

The microscopy technicians in primary health institutions in Chongqing have good microscopy competency in qualitative diagnosis, but there were deficiencies in the identification of plasmodium species. Township level health institutions in Central China have weaker microscopy than those in other areas.

## Data Availability Statement

The raw data supporting the conclusions of this article will be made available by the authors, without undue reservation.

## Ethics Statement

The study was reviewed and approved by the Ethical Committee of Chongqing center for disease control and prevention. All participants provided written informed consent for the study.

## Author Contributions

LF conceived the study, collected and analyzed the data, and drafted the manuscript. YY and LS-S provided suggestions for improving the quality of the data. TY, ZY, and XJ-R provided the test blood slides and collected the scores. ZS initiated the study. All authors contributed to the writing of the manuscript and approved the submitted version of the manuscript. All authors read and approved the final manuscript.

## Conflict of Interest

The authors declare that the research was conducted in the absence of any commercial or financial relationships that could be construed as a potential conflict of interest.
